# Evidence for the impacts of agroforestry on ecosystem services and human well-being in high-income countries: a systematic map

**DOI:** 10.1186/s13750-022-00260-4

**Published:** 2022-03-17

**Authors:** Sarah E. Castle, Daniel C. Miller, Nikolas Merten, Pablo J. Ordonez, Kathy Baylis

**Affiliations:** 1 Department of Natural Resources and Environmental Sciences, University of Illinois Urbana-Champaign, Urbana, IL 61801 USA; 2 Keough School of Global Affairs, University of Notre Dame, Notre Dame, IN 46556 USA; 3 John Glenn College of Public Affairs, The Ohio State University, Columbus, OH 43210 USA; 4 Inter-American Development Bank, Washington, D.C 20577 USA; 5 Department of Geography, University of California Santa Barbara, Santa Barbara, CA 93106 USA

## Abstract

**Background:**

Agroforestry bridges the gap that often separates agriculture and forestry by building integrated systems to address both environmental and socio-economic objectives. Existing empirical research has suggested that agroforestry—the integration of trees with crops and/or livestock—can prevent environmental degradation, improve agricultural productivity, increase carbon sequestration, and support healthy soil and healthy ecosystems while providing stable incomes and other benefits to human welfare. However, the extent of the literature supporting or refuting these claims has not been well documented. This study addresses this research gap by collating and describing the evidence for the impacts of agroforestry on ecosystem services and human well-being in high-income countries and presents the characteristics and gaps in the literature.

**Methods:**

We searched 5 primary databases and 24 organizational websites using a pre-defined search string designed to capture articles relating agroforestry practices and policy interventions to outcomes in high-income countries. Searches included peer-reviewed and grey literature published in the English language between January 1990 and June 2020. We screened the identified articles for inclusion or exclusion in two stages: title/abstract and full text. We extracted data from articles included at the full-text stage to form the map and associated database. For inclusion, the study in question must have assessed the impacts of the deliberate promotion and/or actual integration of woody perennials (trees, shrubs, etc.) with agricultural crops and/or animals.

**Results:**

Our search returned 31,852 articles of which we included 585 primary articles, 6 ongoing primary articles, and 41 systematically conducted literature reviews. The articles spanned three decades and 31 countries. The most studied practices are on linear boundary plantings (hedgerows, shelterbelts, windbreaks, and riparian buffers) and silvopasture systems. The most studied outcome is regulation and maintenance of physical, chemical, and biological conditions as an ecosystem service, followed by agricultural yield and mediation of waste/toxics/other nuisances (nutrient runoff and carbon storage).

**Conclusions:**

Results highlight key evidence gaps and areas where research has concentrated. Knowledge on the impacts of specific policy interventions to promote agroforestry remains scarce. The impacts of actual agroforestry practices are more well-studied, but the kinds of practices studied are limited, with most research focusing on two-component systems consisting of a simple tree configuration with one crop or livestock species, such as shelterbelts, windbreaks, and hedgerows, riparian buffers, and scattered trees on farms with crops and/or livestock. Regulating ecosystem services outcomes are by far the most studied, followed by agricultural productivity (an aspect of provisioning ecosystem services), while evidence on human well-being remains limited. We also found geographic biases, with little to no evidence for many countries. These biases suggest the strong need for further research to build the evidence base on agroforestry across high-income countries. The results can inform future research and policy decisions by making the evidence easily accessible and highlighting knowledge gaps as well as areas with enough evidence to conduct further systematic review.

**Supplementary Information:**

The online version contains supplementary material available at 10.1186/s13750-022-00260-4.

## Background

Agroforestry has risen to prominence as a land-use strategy to help address global climate change and provide other environmental, economic, and social benefits. Agroforestry is promoted for its potential for carbon sequestration, soil erosion and runoff control, and improved nutrient and water cycling, as well as for offering socio-economic benefits and greater agricultural productivity [1–10]. The potential for agroforestry to provide sustainable production, improve food security, increase water quality, combat climate change and biodiversity loss, and reduce poverty, also provides an opportunity to advance the UN 2030 Sustainable Development Goals (SDGs) [2, 11]. Given these diverse potential benefits, agroforestry has seen both an increase in policy support and in scholarly attention in high-income countries (HICs) over the past several decades [4, 12–14]. Evidence of the socio-economic and biophysical impacts of various agroforestry policy interventions and practices in HICs spans many disciplines and addresses a broad range of outcomes, thus creating the need to synthesize the evidence to facilitate knowledge uptake and exchange. To address this research need, we developed a systematic map (SM) showing the evidence of the impacts of agroforestry practices and policy interventions on ecosystem services and human well-being in all HICs published over the last three decades (January 1990–June 2020).

This systematic map therefore aims to assemble the research showing the impacts of agroforestry practices and policy interventions in HICs, with the goal of providing an evidence map of the literature to aid researchers and policymakers in developing strategies for future research initiatives and policy formation. These HICs, as defined by the World Bank [15], are located in North America, Europe, East Asia and the Pacific, Latin America and the Caribbean, and the Middle East, and are listed in Additional file 1. We focus on HICs for four main reasons. First, HICs are primarily located in temperate climates, as opposed to low- and middle-income countries (L&MICs), which are primarily located in tropical and subtropical climates. This contrast across climate zones means that different agroforestry practices and policy interventions are likely relevant in HICs compared to L&MICs. Second, the wealth in high income economies may pose different opportunities and constraints for agroforestry practices and policy interventions. Third, agroforestry research and practice in HICs generally lag behind L&MICs, so focusing on HIC agroforestry can reach an important audience to advance practice and policy in these important regions. Finally, this SM is designed to complement our parallel systematic map on the impacts of agroforestry in L&MICs, which was funded by 3ie (https://www.3ieimpact.org/) only for L&MICS [16].

By mapping the existing evidence on the impacts of agroforestry practices in HICs on ecosystem services and human well-being, we create an easily navigable database of relevant research on this topic and enable a clearer picture of key areas of interest for further research. The results encompass research from all HICs, which will allow researchers and policymakers to utilize knowledge gained from different contexts around the globe.

Agroforestry is defined as the intentional integration of woody vegetation, such as trees and shrubs, with crops and/or livestock simultaneously or sequentially on a land management unit at any scale (e.g., plot, farm, landscape, etc.) [17]. This integration is often intended to diversify production systems to create environmental, economic, and social benefits through complementary interactions between the system components [17–19]. The general types of agroforestry include agrisilviculture (also called silvoarable, defined as trees integrated with cropping systems), silvopasture (trees integrated with livestock systems), agrosilvopasture (trees integrated with both crops and livestock as a system), forest farming (crop or livestock production within a forested area), urban agroforestry (urban agroforests or urban forest gardens, defined as integrating trees with crops near the homestead or in urban areas), and other types, such as integrating trees in fisheries or beekeeping operations [18]. Common agroforestry practices are presented in Table 1.

**Table 1:** Classification of agroforestry systems and specific practices. Definitions are drawn from [18, 20–22]

Land use and agroforestry practice		Brief Description
Agrosilviculture / Silvoarable	Trees integrated in crop fields (multipurpose trees)	Trees intercropped with annual or perennial crops; trees randomly or systematically planted in cropland for the purpose of providing fruit, fuel wood, timber, and other services
	Hedgerows, shelterbelts, and windbreak systems	Trees as fences around plots and/or an extended windbreak of living trees and shrubs established and maintained to protect farmlands
	Alley-cropping systems	Rows of trees with a companion crop grown in the alleyways between the rows
	Improved or rotational fallow	Land resting system using trees and shrubs to replenish soil fertility and potentially yield economic benefits, in rotation with crops as in traditional shifting cultivation
	Riparian buffer strips	Areas along rivers and streams planted with trees, shrubs, and grasses to protect water quality
Silvopasture	Trees/shrubs on pasture (multipurpose trees)	Trees intercropped on pastures; trees randomly or systematically planted on pasture for the purpose of providing fruit, fuel wood, timber, and other services. Also used for forage/fodder and animal production
	Meadow orchards	Orchards, including fruit orchards, olive groves, vineyards, and fruit-bearing shrubs, which are grazed or sown with pastures
	Hedgerows, shelterbelts, and windbreak systems	Trees as fences around plots and/or an extended windbreak of living trees and shrubs established and maintained to protect farmlands and animals and/or provide fodder
Agrosilvipasture	Integrated production of animals (meat and dairy), crops, and wood/fuelwood	Production of crops, animal/dairy, and wood products within the same land area, including around homesteads
Forest Farming	Forest farming	Forested areas used for production or harvest of naturally standing specialty crops for medicinal, ornamental, or culinary uses
	Forest grazing	Forested areas with the understory grazed as a means of providing forage for animal production
Urban and Periurban	Urban agroforests/ urban forest gardens	Combining trees/shrubs with vegetable production usually associated with periurban or urban areas
Agroforestry including insects/fish	Entomoforestry	Production combining trees and insects (e.g., bees for honey and trees)
	Aqua-silvo-fishery	Trees lining fishponds, tree leaves being used as 'forage' for fish

Agroforestry systems include a broad range of practices and configurations, ranging from sparsely retaining trees on a pasture to complex multistrata food forest systems containing dozens of species. A farm may have a range of different agroforestry practices that combine into more complex agroforestry systems, which can provide differing impacts on ecosystem services and human well-being. Additionally, the scale at which agroforestry practice and its impacts are considered (plot, farm, landscape, etc.) can greatly affect the measurement of ecosystem services and human well-being. For example, many regulating ecosystem services may only manifest at the landscape scale rather than at the farm scale where the agroforestry system is located. Agroforestry landscape (e.g., a region with multiple farms practicing agroforestry) may provide very different impacts than a single agroforestry plot. We include studies that consider any combination of agroforestry practices and any scale of analysis, to be as broad and inclusive in the SM as possible.

Agroforestry research began with the study of traditional practices of local populations around the world, which formed the basis for conducting more rigorous experimental research [23]. As agroforestry research developed, researchers found a high potential for agroforestry to address many current environmental and social concerns, such as climate change and food security [23]. From this knowledge base, agroforestry advocates began pushing for the establishment of policies and programs to support the integration of trees on agricultural lands.

We further define several types of policy interventions that may be used to promote any one or more of these agroforestry practices. Agroforestry policy intervention types are described in Table 2, and they represent types of support policymakers could provide to promote adoption of one or more of the agroforestry practices described in Table 1. This SM aimed to identify research related to the impacts of such agroforestry policy interventions on the ecosystem services and human well-being outcomes of interest. We therefore did not include studies that only considered adoption, but rather those that included the impacts of the policy intervention on one or more ecosystem services or human well-being outcomes. We indicate whether a study is an impact evaluation of an agroforestry-related policy intervention or is an evaluation of the effects of an agroforestry practice.

**Table 2:** Classification of policy interventions to promote agroforestry, as presented in [16, 24, 25]

Policy intervention type	Description and examples
Farmer capacity development	Efforts focus on enhancing farmer knowledge and/or skills relevant to agroforestry practice, e.g., setting up and managing tree nurseries; tree planting and management techniques; and seed collection and propagation. Such interventions can involve the provision of training, extension and other advisory services, and specific technical information, as well as the setting up of demonstration sites, running of participatory trials and other modes of participatory action learning
Material support	Efforts to facilitate farmer access to quality and desired tree/shrub seedlings/seeds required to pursue prioritized agroforestry practices. Such interventions often entail the direct provision of seedlings/seeds to farmers but can also involve linking farmers to relevant suppliers and/or enhancing the ability of existing or new suppliers to supply participating farmers with quality and desired tree germplasm
Incentive provision	Interventions of this type seek to motivate farmers to plant trees and practice agroforestry through the provision of incentives. Examples include paying farmers for planting and caring for trees on their farms in exchange for desired ecosystem services (e.g., carbon sequestration) and buyers offering premiums to farmers for agricultural commodities produced under certain conditions (e.g., via certification schemes for products such as shade grown organic coffee)
Community-level campaigning and advocacy	Interventions of this type can also involve the provision of information about the benefits of trees and agroforestry and/or the provision tree seedlings/seeds, but this type is distinct from the first two types. The main objective is to motivate, including through social pressure, community members to plant trees on their farms and/or pursue specific agroforestry practices. Campaigning and advocacy may be done through radio and/or community meetings, speeches, and drama and may involve a mass community effort to plant trees, for example, on a specific day of the year
Market linkage facilitation	Interventions of this type focus on efforts to enhance potential returns from agroforestry to encourage adoption. This could be through linking producers to and/or brokering new and/or improving existing contractual arrangements with buyers. Other examples include the collective marketing of agroforestry products and/or interventions to stimulate demand for a given agroforestry product, e.g., pawpaw fruit
Institutional and policy change	Interventions of this type involve reforming and/or putting in place new polices, laws, regulations, and institutions more broadly to facilitate greater uptake of and benefits from agroforestry. Such efforts are designed to address existing policy and institutional constraints such as, for example, prevailing forestry regulations—designed for forest management areas—that may frustrate smallholder efforts to grow some high-return tree species or insecure land tenure that may similarly deter long-term adoption of tree plantings

There are abundant examples of agroforestry practices throughout the world, but the initiatives to create policies and programs that formalize and promote agroforestry are relatively new in most HICs. International groups, such as World Agroforestry (ICRAF), have invested in agroforestry projects in L&MICs for decades (emerging in the 1960s and 1970s) as a solution to address environmental degradation, boost food security, and contribute to a range of other development policy objectives [2, 26]. By contrast, agroforestry policy in the United States (USA), for instance, was first introduced in the mid-1980s (though promotion of windbreaks to reduce soil-erosion during the 1930s Dust Bowl era is a precursor for the specific practice), with more formalized agroforestry policy emerging only in the 1990s with the Forest Stewardship Act of 1990 establishing a Center for Semiarid Agroforestry (renamed the National Agroforestry Center in 1994, broadening its scope to include the entire country). Similarly in the European Union (EU), agroforestry promotion began in the early 1990s with the 1992 reform of the EU Common Agricultural Policy (CAP), which formerly encouraged practices that discouraged farmers from integrating trees on farms [27]. Only within the last decade has there been any significant uptake of agroforestry projects in HICs in the context of institutionalized support for agroforestry as an alternative land use approach to address conservation and sustainable agricultural development objectives [13, 28–30].

There is evidence showing that agroforestry has the potential to offer many ecological benefits – environmental, economic, and social – which can incentivize landowners to adopt such practices [5, 9, 31–34]. There is a growing interest in the potential of agroforestry and an increasing awareness of the role agroforestry can play in creating a diversified, multi-dimensional farming system [12, 13, 31, 35, 36]. Nevertheless, viewed in broader perspective, the integration of agroforestry into practice is still relatively low. For instance, the United States Department of Agriculture (USDA) estimates that agroforestry is applied on less than 1% of agricultural land with the potential for agroforestry through USDA assisted programs [21]. This SM will therefore provide important evidence synthesis that may support initiatives to disseminate agroforestry knowledge and promote the implementation of agroforestry in suitable areas as an alternative land use strategy across different HIC contexts. Additionally, it will help to find evidence of potential tradeoffs that come with the establishment of agroforestry practices.

To our knowledge, this is the first systematic map covering agroforestry so broadly and with a global scope, notably when combined with our parallel L&MIC agroforestry systematic map, hereby referred to as the L&MIC evidence and gap map (EGM) [16]. However, there have been several other literature reviews and systematic reviews that described agroforestry literature. Recent literature reviews have given overviews of the evidence for the impacts of agroforestry on ecosystem services and environmental benefits [9], climate change adaptation and mitigation [37], carbon sequestration [5], biomass production [5, 7, 32], soil health [7, 35], and food production [7]. These did not, however, follow systematic review methods, which limits the repeatability, objectivity, comprehensiveness, and transparency of the review methodology. Therefore, these studies are generally considered less rigorous, and the results of these studies may be less objective or thorough. Several recent efforts have sought to systematically map and review aspects of agroforestry. Notably, one group mapped the evidence on agroforestry impacts on biodiversity and ecosystem services across Europe [3, 38]. Recently, another group mapped the evidence on the impacts of temperate agroforestry with livestock (silvopasture or forest grazing) on farm productivity, water quality and quantity, air quality, soil erosion, and enterprise economics [31]. Others include aspects of agroforestry, such as a systematic map on the impacts of vegetated strips—including windbreaks, hedgerows, and shelterbelts—on nutrients, pollutants, socioeconomics, biodiversity, and soil retention in boreo-temperate systems [39]. Another study mapped the impacts of Ecological Focus Area options (including agroforestry) in European farmed landscapes on climate regulation and pollination services [40]. Finally, we note that a systematic map of the effects of nature conservation on human well-being [41] and one on forests and poverty globally [42] include some studies on the impacts of agroforestry. We are not aware, however, of any previous effort to systematically map evidence on the impacts of agroforestry policy interventions and practices on the broad range of ecosystem services and human well-being outcomes across all HICs. Lack of such evidence synthesis constrains the ability of policymakers, practitioners, and researchers to make effective decisions relating to agroforestry.

There are two primary audiences for this SM. First, we expect that researchers on agroforestry and broader sustainability issues will use the results to inform further investigations on these topics, including new empirical research, as well as systematic reviews of specific linkages and further evidence synthesis. Results should be of wide interest to researchers in a range of institutions, particularly national programs (USDA, AGFORWARD, etc.), national and regional agroforestry associations and extension programs, and universities. The second main anticipated audience is decision-makers for whom agroforestry is already or potentially of interest. This includes relevant government ministries and agencies, non-governmental organizations (NGOs), and other advocacy and implementing organization staff. These groups of decision-makers can use this SM to guide funding priorities for policies and future research based on evidence. This SM database base provides policymakers with a resource to make evidence-based policy decisions for agroforestry initiatives.

### Theory of change

There are several pathways through which agroforestry might deliver ecosystem services and human well-being. Once a farmer adopts agroforestry, they may see improved soil health and other ecosystem services, such as improved water infiltration and decreased nutrient runoff, which then increase crop productivity or reduce production costs and, therefore, increase returns. Some agroforestry farmers may find that increased use and availability of tree/shrub fodder and shade lead to increases in animal product production and returns. Selling other agroforestry products such as timber, firewood, fruit, and nuts can increase and diversify income and food sources [12, 37, 43–45]. Together, these outcomes are expected to bolster resilience to shocks, as well as boost overall farmer income and food security.

To support farmers to practice agroforestry, policy interventions may be necessary to support such practices that can deliver desired ecosystem services and potentially increase profitability of these systems. For example, carbon markets can pay farmers for planting carbon sequestering trees and increase the profitability of the systems through the payments. Subsidies and tax breaks for agroforestry farmers can also increase the economic profitability of these practices and preserve existing traditional agroforestry systems (e.g., dehesas/montados). Other interventions, like education and extension, or providing access to appropriate tree germplasms, can support farmers to implement appropriate practices effectively and deliver improved ecosystem services. These changes may have differential effects depending on gender, socio-economic status, race/ethnicity, or education/literacy level, particularly when considering the impacts of policy interventions intended to support agroforestry.

However, it is worth noting that there are potentially negative tradeoffs to agroforestry, such as a reduction in area of crop production and negative tree-crop interactions [46–48]. In some cases, agroforestry can reduce production and profitability, while delivering desirable ecosystem services. In such cases, policy interventions, such as payments for ecosystem services or increasing market value of products through consumer-recognized certification schemes, can offset the economic losses and increase profitability while delivering ecosystem services.

### Stakeholder engagement

In preparing the protocol for the parallel L&MIC agroforestry EGM, on which our protocol was based, we coordinated with colleagues involved in two related evidence maps [41, 42] and our team engaged with an advisory group comprised of 3ie (https://www.3ieimpact.org/) members, donor agency staff, International Development Coordinating Group (IDCG) members and other evidence synthesis experts, World Agroforestry Center (ICRAF) scientists and other agroforestry subject experts. These consultations informed our systematic approach and helped define several important terms for our search string, which were proposed in our protocol for this SM. The HIC SM protocol was presented as a poster presentation at the Green Lands Blue Waters conference in Madison, Wisconsin in November 2017 and discussed with interested agroforestry experts. Feedback and suggestions given to the authors were incorporated into the HIC protocol. The preliminary results of the HIC SM was presented as an oral presentation at the 4^th^ World Congress on Agroforestry in Montpellier, France in May 2019 and at the 16^th^ North American Agroforestry Conference in Corvallis, Oregon in June 2019 during which feedback on this work was sought.

## Objective of the review

The primary aim of this systematic map is to identify, map, and describe existing evidence on the effects of agroforestry practices and policy interventions in HICs.

The primary research question of this systematic map is: *What evidence exists on the effects of agroforestry practices and policy interventions on ecosystem services and human well-being in HICs?*

To address these research questions, the scope is defined by the Population, Intervention/ Practice, Comparator, and Outcome (PICO) components to be examined as presented in Table 3.

**Table 3:** Elements of the Agroforestry Systematic Map

Population (Subject)	Intervention or Practice	Comparators	Outcomes
Farmers and/or farmland in high-income countries/lending groups	Adoption or implementation of one or more of the defined agroforestry practices or policy interventions	Control site without agroforestry; or, before-after time-series comparison on same site	Positive, negative, or neutral effects on ecosystem services or human well-being

## Methods

This SM was conducted according to a previously published protocol [49] and it followed the Collaboration for Environmental Evidence guidelines and standards for evidence synthesis in environmental management [50]. The map conforms to Reporting standards for systematic evidence syntheses (ROSES) [51] (see Additional file 2). Any deviations from the original protocol are noted.

### Deviations from the protocol

We deviated from the protocol by extending our search through June 2020, instead of through mid-2018 as originally planned. We decided to update the SM through mid-2020 based on the time required to complete the mapping from 1990 through mid-2018.

We also deviated in our framing and coding strategy for our outcomes of interest based on suggestions from reviewers. Specifically, we remove the redundant category of agricultural productivity since both yield and profitability are captured under the ecosystem services and human well-being categories.

### Search strategy

The methods for the searches, screening, and inclusion criteria closely follow those used for the parallel L&MIC EGM [16, 52], with a few modifications to adapt the process to account for differences between HIC and L&MIC concepts of agroforestry. Specifically, we modified the search string to include additional agroforestry terms, impact terms, and HIC terms (instead of L&MIC terms). We made these changes based on our literature review and test list retrieval performance assessment, as documented in [49]. Furthermore, we note that the L&MIC EGM has a stronger emphasis on agroforestry interventions since it was conducted through the International Initiative for Impact Evaluation (3ie) (https://www.3ieimpact.org/), which focuses more on synthesizing evidence on the impacts of interventions. This SM intends to capture studies on the impacts of both agroforestry interventions as well as agroforestry practices in general, without placing emphasis on one or the other.

We conducted a comprehensive search using multiple sources to best capture an unbiased representation of existing literature. The searches were carried out on multiple bibliographic databases and on relevant organizations webpages (grey literature sources). Articles from January 1, 1990, through June 1, 2020, were included in the search. We conducted our first search through October 1, 2018, and we conducted a follow-up search on June 1, 2020. We begin the study period in 1990 as this was a pivotal point for environmental issues gaining traction in global policy and when environmental aid investment escalated, following the Earth Summit in 1992 and the Framework Convention on Climate Change and the Convention on Biological Diversity [53]. The early 1990s was roughly the time that HICs saw increased support for agroforestry and other approaches designed to further environmental goals, as discussed earlier. The search was done through use of search engines, based on key words within the identified databases. When such a strategy was not possible (e.g., for some topical databases and organizational webpages), hand searches were performed to extract all potentially relevant articles. A detailed assessment of retrieval performance is provided in [49]. Due to resource constraints, we only included English language articles, which places limits on the comprehensiveness of this study. The databases and search terms are described in full in [49] and Additional file 3.

The bibliographic databases that were searched for publications were:

- SCOPUS
- EBSCO: Agricola, Econlit
- Web of Science: Core Collection
- CAB Abstracts and Global Health
- AGRIS

The full list of relevant organization websites (grey literature sources) searched is described in Table 4 and Additional file 4.

**Table 4:** List of websites from relevant organizations

Organization	Website
AGFORWARD	https://www.agforward.eu/
Agriculture Research Service (USDA)	https://www.ars.usda.gov/
Association for Temperate Agroforestry	http://www.aftaweb.org/about/afta.html
The Center for Agroforestry at the University of Missouri	http://www.centerforagroforestry.org/
Collaboration for Environmental Evidence	www.environmentalevidence.org
Conservation Evidence	http://www.conservationevidence.com
European Commission Agriculture and Rural Development	http://ec.europa.eu/agriculture/
European Agroforestry Federation (EURAF)	https://euraf.isa.utl.pt/welcome
European Environment Agency	http://www.eea.europa.eu/
Farm Woodland Forum	http://www.agroforestry.ac.uk/
Food and Agriculture Organization (FAO)	http://www.fao.org
Global Forest Information Service (GFIS)	https://www.iufro.org/science/gfis/
IDEAS RePEc (Research Papers in Economics)	https://ideas.repec.org
IEEP	http://www.ieep.eu/
International Union for the Conservation of Nature	http://www.iucn.org
National Agroforestry Center (USDA)	https://www.fs.usda.gov/nac/index.shtml
Natural Resources Conservation Service (USDA NRCS)	https://www.nrcs.usda.gov/wps/portal/nrcs/site/national/home/
NERC Open Research Archive	https://nora.nerc.ac.uk/
New Zealand Grassland Association (NZGA)	https://www.grassland.org.nz/
SAFE: Silvoarable Agroforestry For Europe	http://www1.montpellier.inra.fr/safe/english/index.htm
Sustainable Agriculture Research & Education (SARE)	https://www.sare.org/
United Nations Environment Programme (UNEP)	http://www.unep.org
UK Department for Environment Food and Rural Affairs	https://www.gov.uk/government/organisations/department-for-environment-food-rural-affairs
World Agroforestry Center	http://www.worldagroforestry.org/

### Article screening

The first author led the screening of the retrieved articles, and two research assistants helped with the screening. We imported the records from academic databases into our data management software (EPPI-Reviewer 4), and we used the built-in tool to aid in removing duplicates. The grey literature and additional searches were imported into and managed in Microsoft Excel due to resource limitations and reference format incompatibility with EPPI-Reviewer 4. We then first screened the records at the title and abstract level, excluding articles that did not meet our criteria for study country/lending group, publication year, study type, and relevant agroforestry practice or intervention.

As with other areas of science [54], many articles used titles and abstracts that did not emphasize our area of interest but were possibly relevant, or were otherwise unclear whether they were relevant, making it difficult to determine whether a paper met the inclusion criteria at the title/abstract stage. In these cases, we had to review the full text. For the initial consistency check, we double screened a small subset of 100 training articles at the title and abstract stage and then use the approach in [55] for securing agreement among coders. This training set consisted of 100 articles randomly selected from our search in Web of Science. The inter-rater reliability was calculated using a Kappa statistic for all articles double screened at title and abstract levels [56]. The result of our Kappa test was 0.66, with over 83% agreement between reviewers. If the Kappa test agreement fell below 0.6, indicating moderate agreement, an additional reviewer would have been consulted and an additional set of 100 test articles would have been screened by all reviewers, as in [40, 57]. However, given that we achieved moderate agreement, we instead had a detailed discussion about any discrepancies until we agreed on an appropriate coding strategy. As a continued consistency check, a subset of the articles was screened by two reviewers throughout the title and abstract screening stage and the full text screening and data extraction stage, as described below.

The online literature review and reference management software EPPI-Reviewer 4 [58] was used to upload potentially eligible titles and abstracts for candidate articles identified through the search strategy. Grey literature was documented in Microsoft Excel. During the screening process, when a rater was uncertain about study eligibility, the relevant study was marked for a second opinion and screened by a second rater. The lead reviewer checked the consistency among the members of the mapping team periodically throughout the title and abstract screening phase and at the full-text screening stage for a subset of articles. At both the title and abstract screening phase and the full-text screening and data extraction stage, a subset of approximately 10% of the articles were assessed by at least two reviewers. This consisted of weekly random spot-checking for inter-rater agreement on the inclusion decisions and data extraction of screened studies as well as a thorough team discussions on any discrepancies and inclusion uncertainty among members of the mapping team. Where there was an inconsistency or disagreement between reviewers the study was marked as “Re-evaluate” in EPPI-Reviewer 4 and was discussed by reviewers to reach agreement.

### Study eligibility criteria

Given that this map is meant to offer a resource for decision-makers, as well as identify gaps and well-researched areas in the current evidence base, it includes both primary studies and systematically conducted reviews. All included studies must explicitly examine the outcomes of specific agroforestry practices or agroforestry policy interventions. Furthermore, they must use a comparator, which may be temporal, spatial, between group, or some combination of these. We excluded theoretical or modeling studies (unless they included a relevant empirical example with a design that met inclusion criteria), and editorials and commentaries. Experimental trials managed by researchers were not included due to time and resource constraints and since the population of interest for this systematic map is farmers and farmer’s land. Experimental off-farm trials, however, were excluded into a separate bin in EPPI-Reviewer 4 and are listed in Additional file 6 as a base for future work and synthesis. On-farm field trials were included if all other eligibility criteria were met.

Four kinds of studies were included: (1) quantitative impact evaluations, (2) systematically conducted reviews (including systematic maps, systematic reviews, meta-analyses using systematic searches, and ongoing systematic map/review protocols), (3) farmer-implemented field trials that test specific agroforestry techniques and approaches, and (4) observational studies on the effect of agroforestry practices. Detailed descriptions explaining each of these types of studies is given in [49].

The PICO format (population, intervention, comparator, outcome) was used to define the inclusion criteria for this SM as follows.

#### Eligible subject (population)

The subject of interest will be farms and/or the people that live and farm on them that are incorporating any agroforestry practices into their farming system within the high-income countries.

#### Eligible intervention or practice

Eligible articles studied one or more agroforestry practices (Table 1) or agroforestry policy interventions (Table 2). From a policy perspective, it may be especially useful to know what kinds of policy interventions might most effectively promote agroforestry practices to yield desired social-ecological outcomes. However, the evidence on the impacts of agroforestry practices can be essential to informing future policy decisions as well as landowners and other practitioners. In our map, we indicate studies that include an evaluation of an agroforestry-related policy intervention, versus studies that evaluate the impact of only an agroforestry practice without a policy intervention.

#### Eligible comparator

Only studies that used a comparator were considered for inclusion. We considered four types of eligible comparators. First, we considered studies that compared farm(s) or household(s) that practiced agroforestry (identified in Table 1) with those that did not practice agroforestry. Similarly, we considered studies where farm(s) or household(s) exposed to a specific agroforestry policy intervention were compared to those that were not exposed. Second, we considered studies that compared farm(s) or households before and after adopting a given agroforestry practice, or before and after being exposed to a specific agroforestry policy intervention. Third, we considered studies comparing land with agroforestry practice(s) with primary forests, secondary forests, or managed forestry/plantations. Fourth, we considered studies applying a combination of two or more of the above criteria. We did not include studies that only compare agroforestry practices with other agroforestry practices without a non-agroforestry comparator (i.e., studies that only evaluated different implementations of the same agroforestry practice, or studies that only evaluated multiple types of agroforestry practices).

#### Eligible outcomes

We included studies relating to two broad outcomes of interest in this SM: ecosystem services and human well-being.

We define the key outcomes of “ecosystem services” as “the benefits people obtain from ecosystems,” following well accepted definitions from the Intergovernmental Science-Policy Platform on Biodiversity and Ecosystem Services (IPBES). The ecosystem services category comprised of the three broad ecosystem services outcome categories outlined in Table 5: (1) provisioning services, (2) regulation and maintenance (or, regulating and supporting) services, and (3) cultural services. Ecosystem service outcomes are further divided into a number of specific categories following the Common International Classification of Ecosystem Services (CICES) developed by the European Environment Agency [59] and presented in Table 4. CICES builds from the seminal Millennium Ecosystem Assessment [60], The Economics of Ecosystems and Biodiversity [61], and other ecosystem services classification schemes.

**Table 5:** Classification of ecosystem services outcomes in broad and specific categories. Specific categories divide each broad category into main types of output or process [59]

Broad Category	Specific category	Examples
Provisioning	Energy	Biomass-based energy sources (plant and animal)
		Mechanical energy (animal-based)
	Materials	Biomass (e.g., Fiber and other materials from plants, and animals for direct use or processing)
		Water (Surface or ground water for non-drinking purposes)
	Nutrition	Biomass (e.g., cultivated crops, reared animals and their outputs, wild plants and animals and their outputs, etc.)
		Water (e.g., surface or groundwater for drinking)
Regulation & Maintenance	Mediation of waste, toxics, and other nuisances	Filtration/sequestration/storage/accumulation/ Mediation of smell/noise/visual impacts
		Weed and pest control
	Mediation of flows	Mass stabilization and control of erosion rates
		Hydrological cycle and water flow maintenance
		Flood & storm protection
		Ventilation and transpiration
	Maintenance of physical, chemical, biological conditions	Lifecycle maintenance, habitat, and gene pool protection (Pollination and seed dispersal, maintaining nursery populations and habitats)
		Biodiversity
		Pest and disease control
		Soil formation and composition
		Water conditions
		Atmospheric composition and climate regulation
Cultural	Physical and intellectual interactions with environmental settings	Physical and experiential interactions (use of plants and animals)
		Intellectual and representative interactions (scientific, education, heritage/cultural, aesthetic, etc.)
	Spiritual, symbolic, and other interactions with environmental settings	Spiritual and/or emblematic (symbolic, sacred, and religious use of plants and animals)
		Other cultural outputs (existence, bequest of plants and animals)

**Table 6:** Domains and definitions of human well-being outcomes, as presented in [52] (adapted from [41])

Domain	Definition
Income and household expenditure	Total household income and expenditure, farm and non-farm income, employment, employment opportunities, wealth, poverty, savings, payments, loans
Housing and material assets	Shelter, assets owned, access and availability of fuel and basic infrastructure (electricity, water, telecommunications, and transportation)
Food security and nutrition	Physical and economic access to sufficient, safe, and nutritious food that meets dietary needs and food preferences for an active and healthy life (FAO). Usually measured using food consumption, expenditure, prevalence of undernourishment and nutritional status
Health	Physical health, longevity/life expectancy, maternal health, child health, access to health care, occurrence of diseases, mental health
Cultural and subjective well-being	Measures of happiness, quality of life, cultural, societal, and traditional values of nature, sense of home, cultural identity and heritage, spiritual or religious beliefs and/or values
Other	E.g. informal education (i.e. transfer of knowledge and skills); social relations (i.e. interactions between individuals and within and/or between groups); governance (i.e. structures and processes for decision making including both formal and informal rules); land and resource security; freedom of choice and action (i.e. ability to pursue what one values doing and being); adaptive capacity and resilience (i.e. ability to cope with perturbations and take advantage of new opportunities due to social and environmental change, especially climate impacts)

We recognize that recently IPBES has turned to the term “nature’s contribution to people,” to supersede the term “ecosystem services.” We chose to maintain our use of the term “ecosystem services” since we designed our study and published our protocol based on this classification system. The use of the term “ecosystem services” largely, if not fully, covers “nature’s contribution to people,” and we do not believe this choice substantially affects our SM results.

Human well-being is the second set of outcomes of interest. We define “human well-being” as “people’s ability to live a life they value” following the International Union for Conservation of Nature (IUCN) definition. Human well-being and ecosystem services are linked with each other in both directions. For the human well-being outcomes, we adapted the classification published in [41] to identify a set of key policy-relevant domains of human well-being (Table 6). Based on likely policy interests and goals typically articulated by proponents of agroforestry, we focus on five dimensions of human well-being: income and household expenditure, housing and material assets, food security and nutrition, health, and cultural and subjective well-being. We also include the category of “other” which groups studies focusing on the other dimensions of human well-being identified in McKinnon et al. [41]. In this last category, we note any mention of adaptive capacity or resilience, especially with reference to the impacts of climate change. The inclusion of papers related to income and expenditure helped us capture literature on the economic impacts of agroforestry, an important but often overlooked outcome in agroforestry research. While there is some overlap between the outcome categories, specifically the cultural services and well-being outcomes, we used this framework to enable easier searchability of our SM database, and coded papers based on all relevant outcomes studied (Additional file 5).

Studies that focus exclusively on the adoption of a particular agroforestry technique or species without reference to impact were excluded. The primary outcomes are those in the two categories stated above (ecosystem services and human well-being), and secondary outcomes such as adoption and behavior change were only reported in the SM if the study also reported primary outcomes.

In the following sections, we present the outcomes in the SM main matrix in two ways: (1) a simplified typology of broad agroforestry practice/intervention and outcome categories and (2) a more detailed version with the specific agroforestry practice/intervention and outcome categories.

#### Types of settings

Agroforestry practices/interventions and outcomes take place in a range of settings in HICs. We defined HICs, per the World Bank definition of economies, as countries and lending groups with high-income economies. Therefore, our list includes several regions classified as lending groups as well as countries. These settings cover a range of ecoregions and are primarily rural, but they may also be urban areas (e.g., city gardens). We included studies that pertain to both smallholder and large landowners.

### Study validity assessment

This study includes information about type of study design, referring to the types of study design presented above, including quantitative impact evaluations (experimental or quasi-experimental), systematically conducted reviews, on-farm field trials (farmer-managed or researcher-managed), and observational studies on the effect of agroforestry practices. Furthermore, the type of quasi-experimental methods used, if applicable, were documented. This data is not intended to offer an assessment of study quality, but rather provide basic information to get a broad perspective of the type of research being conducted in each area of the typology.

### Data coding strategy

We used a standardized data extraction form to extract descriptive data from all articles meeting our eligibility criteria (Additional file 5). We extracted from each article: (1) bibliographic and funding information, (2) study design and basic information, including the study description, information on the practice/intervention type(s), location, sample size, comparators, and equity focus groups, (3) outcome information, including types of outcomes evaluated, indicator variables, and outcome data notes, and (4) mechanisms for policy interventions (where applicable). A codebook describing in detail the scope of each component of the data extraction form is included as part of Additional file 5.

Meta-data extraction was conducted by three reviewers. Our standard for data extraction, which is fully documented in Additional file 5, was followed by all reviewers conducting data extraction. Given the volume of articles, we did not carry out extensive side-by-side double extraction of data at the full text stage. Instead, we conducted random spot checks of a small percentage (~ 10%) of included articles to ensure consistency between raters. This consisted of weekly random spot-checking for agreement among the mapping team of the data extraction for included studies. We engaged in thorough team discussions on any discrepancies or uncertainties among members of the mapping team. Where there was an inconsistency or disagreement between reviewers, the study was discussed by reviewers to reach agreement.

### Data mapping method

We collated a systematic map database in Microsoft Excel (2016, Version 2110, Build 16.0.14527.20270), providing a searchable spreadsheet of the articles and their coded data. The SM results are presented as a series of statistics and figures. Bar plots are used to summarize frequency data. Heatmaps with study counts shown of the intersections of practices/interventions and outcomes are presented to visualized concentrations and gaps in the evidence base. The summary statistics, frequency tables, maps, and heatmaps were calculated and plotted using R (2021, Version 4.1.3.1073). Following data coding, summarizing, and plotting results, the reviewers discussed and collectively identified areas of knowledge gaps and clusters of higher-quality literature based on their experience from screening full-texts.

## Review findings

### Search results

**Fig. 1:**
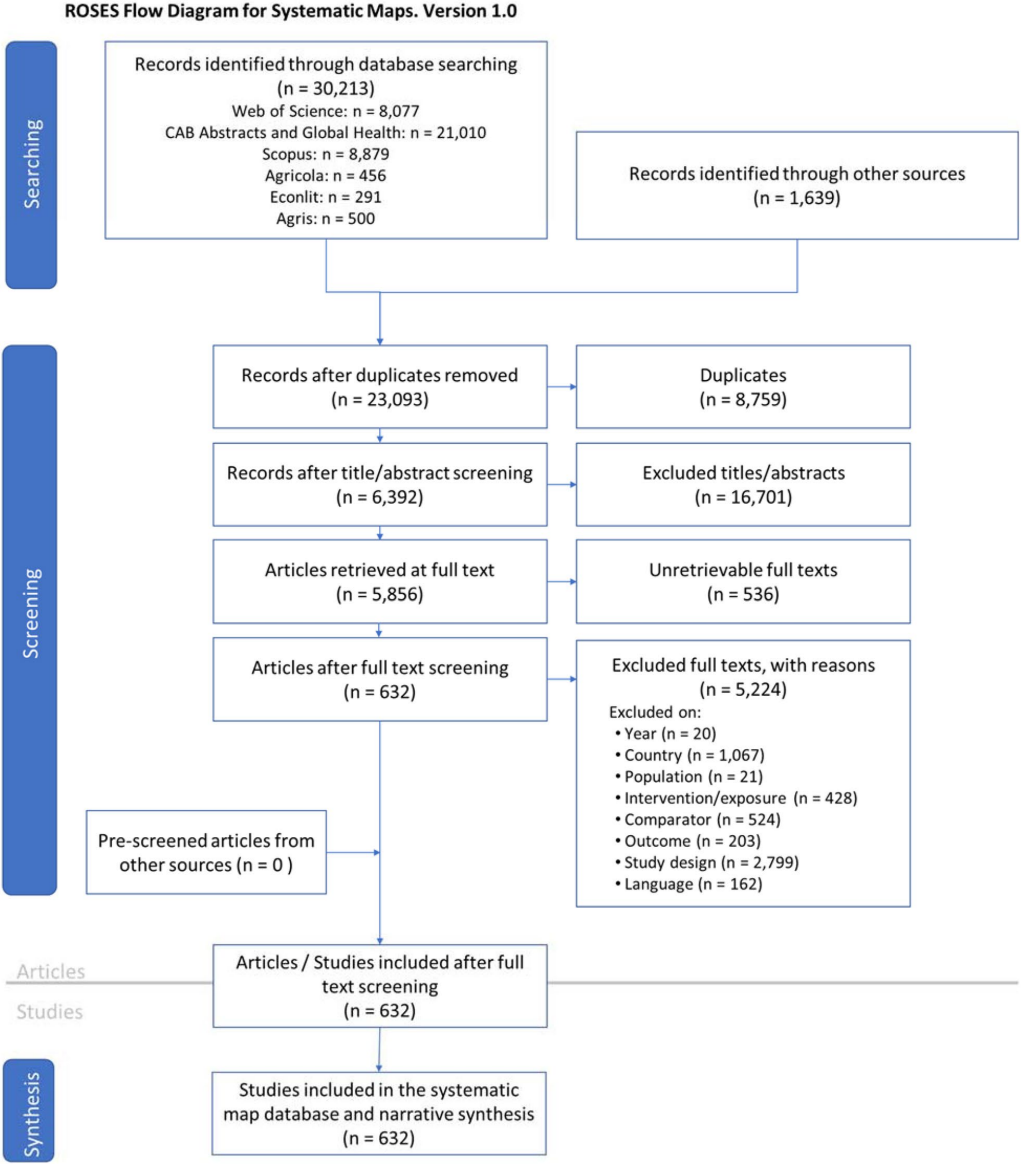
PRISMA Flow Diagram

Figure 1 provides an outline of the search and screening process we used to identify included articles. We identified 31,852 records through our search of academic databases and topical website-based databases (grey literature). We removed 8,759 duplicate articles. After the title and abstract screening, we then attempted to download the 6,329 remaining potentially eligible records for full text screening. We could not find/access 536 of these articles. At the full-text stage, we included 632 articles for data extraction.

Of these 632 included articles, 585 articles present empirical evidence on the impacts of agroforestry practices, of which 33 report evidence on the impacts of agroforestry interventions (only one of which is an impact evaluation), 6 articles are ongoing, and 41 articles are systematically conducted reviews. The six ongoing articles were study reports from the USDA Sustainable Agriculture Research and Education grant program, and we do not include these articles in the charts and discussions below due to insufficient information. Ten of these systematically conducted reviews had protocols associated with them. Three were protocols for reviews in progress. All included articles were published by the time of our search. We also identified approximately 780 additional articles as field trials potentially relating to agroforestry practices. These articles were excluded and not reviewed further; however, they comprise an evidence base of potential interest for further study. Additionally, we captured 690 simulation/modeling articles of potential interest evaluating the potential outcomes or design optimization of agroforestry systems. The reasons why articles were excluded at each stage is presented in Fig. 1. The full list of unretrievable articles and articles excluded at full text is provided in Additional file 6.

### Characteristics and trends of the evidence base from primary studies

The number of studies shown in each distribution chart refers to the total number of studies falling under each domain presented. Individual articles may be classified under multiple domains. For instance, if a study examines the impacts of multiple practices, that study would add to the count for each practice associated with that paper. The sum of studies for each figure may therefore be greater than the number of unique articles associated with that figure.

The included primary studies compare agroforestry practices against other land use practices, such as conventional agricultural or forestry, for at least one of the outcome categories we defined. As such, they represent the existing evidence relating different agroforestry practices and social-ecological outcomes. We identified 585 completed primary articles that included study of at least one agroforestry practice and at least one relevant outcome and these are listed in Additional file 5.

There were six publication types for primary articles included in this SM. Most of the practice articles included are journal articles (n = 531, 91%). Of the remaining primary articles, five are book chapters (< 1%), 25 are conference proceedings (4%), 17 are organization reports (3%), three are technical reports (< 1%), and four are dissertations/theses (< 1%).

**Fig. 2:**
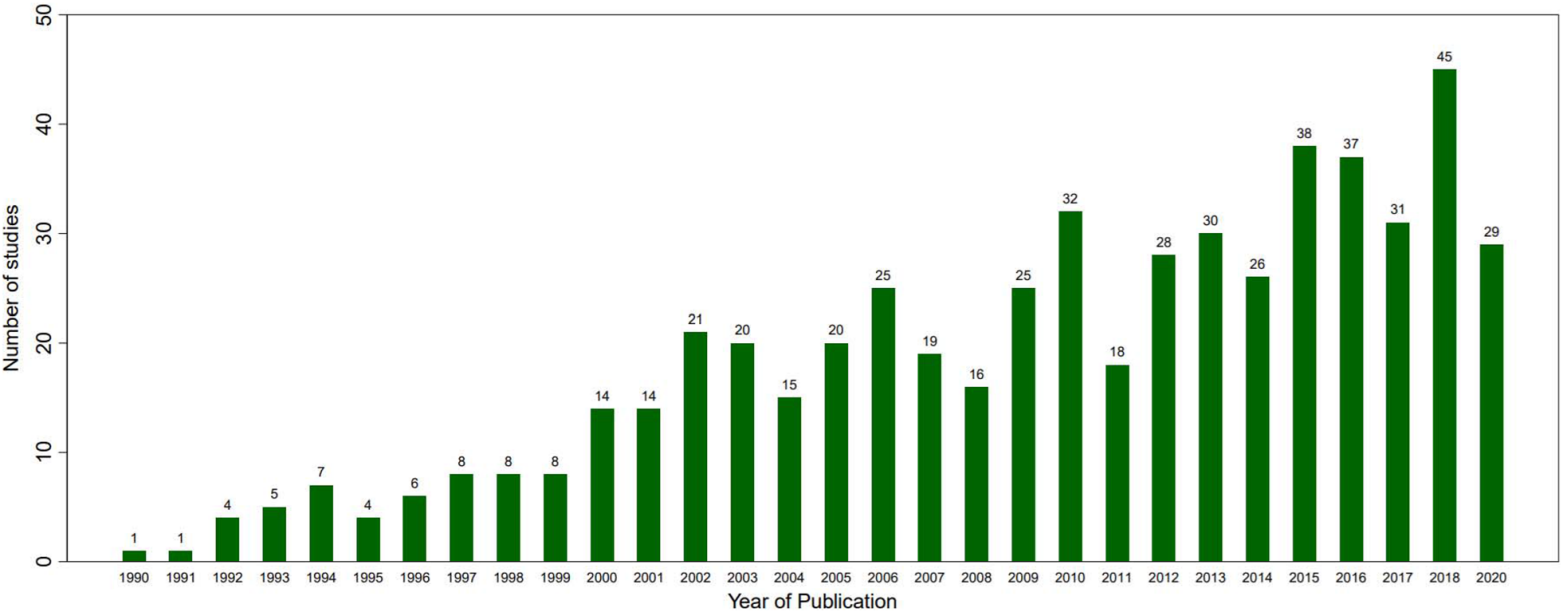
Empirical evidence for agroforestry practices/policy interventions (primary studies) by publication date

The trend of number of publications by year from 1990 to 2020 is presented in Fig. 2. There is an upward trend over the past three decades in publications, with over five times as many articles published in the decade from 2010 to 2019 than in the decade from 1990 to 1999. The data for 2020 only extends through the first half of the year to June 1, 2020, the cutoff for our search.

Most of the primary studies (n = 469, 80%) were classified as observational studies. There were 76 studies (13%) that were experimental (conducted on-farms), and 30 before-after studies (5%). Finally, there were 37 perception/survey type studies (6%). Overall, there was a concentration of observational studies within the evidence base.

**Fig. 3:**
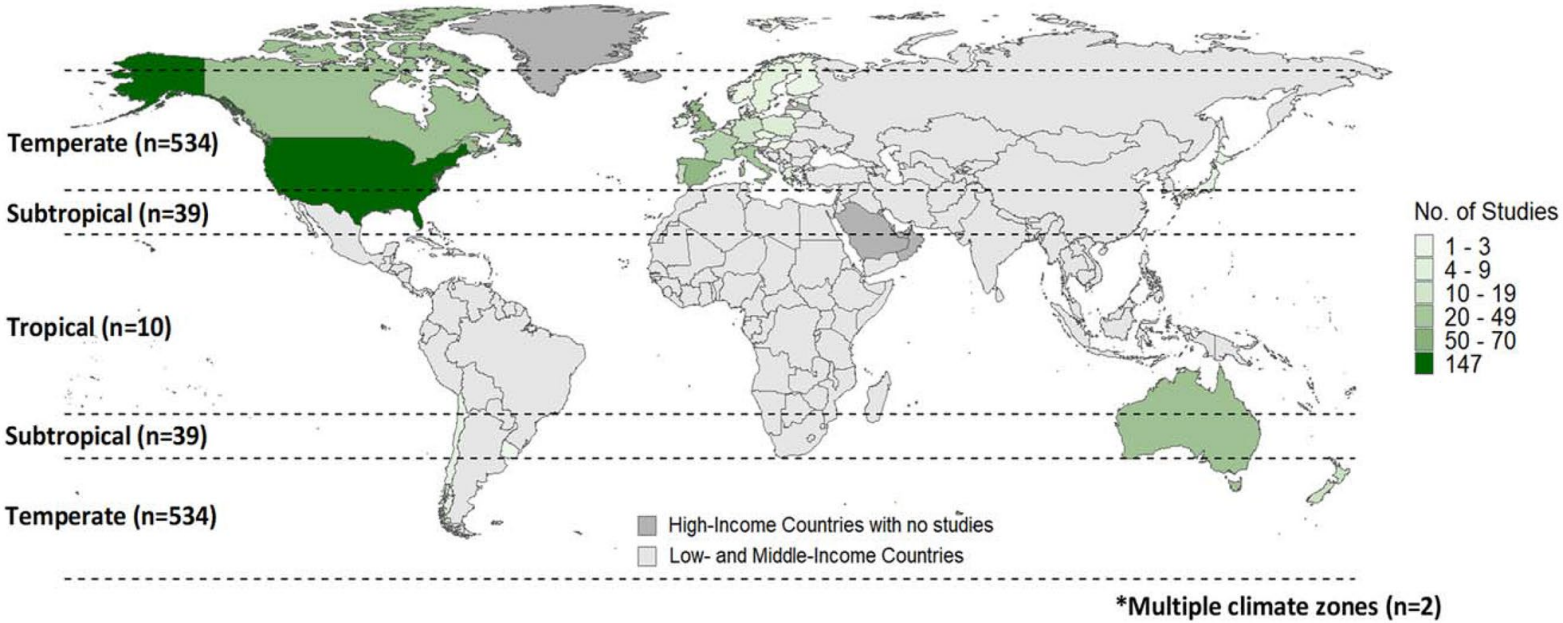
Distribution of primary studies by country/lending group, per the World Bank classification system, and climatic zone

**Fig. 4:**
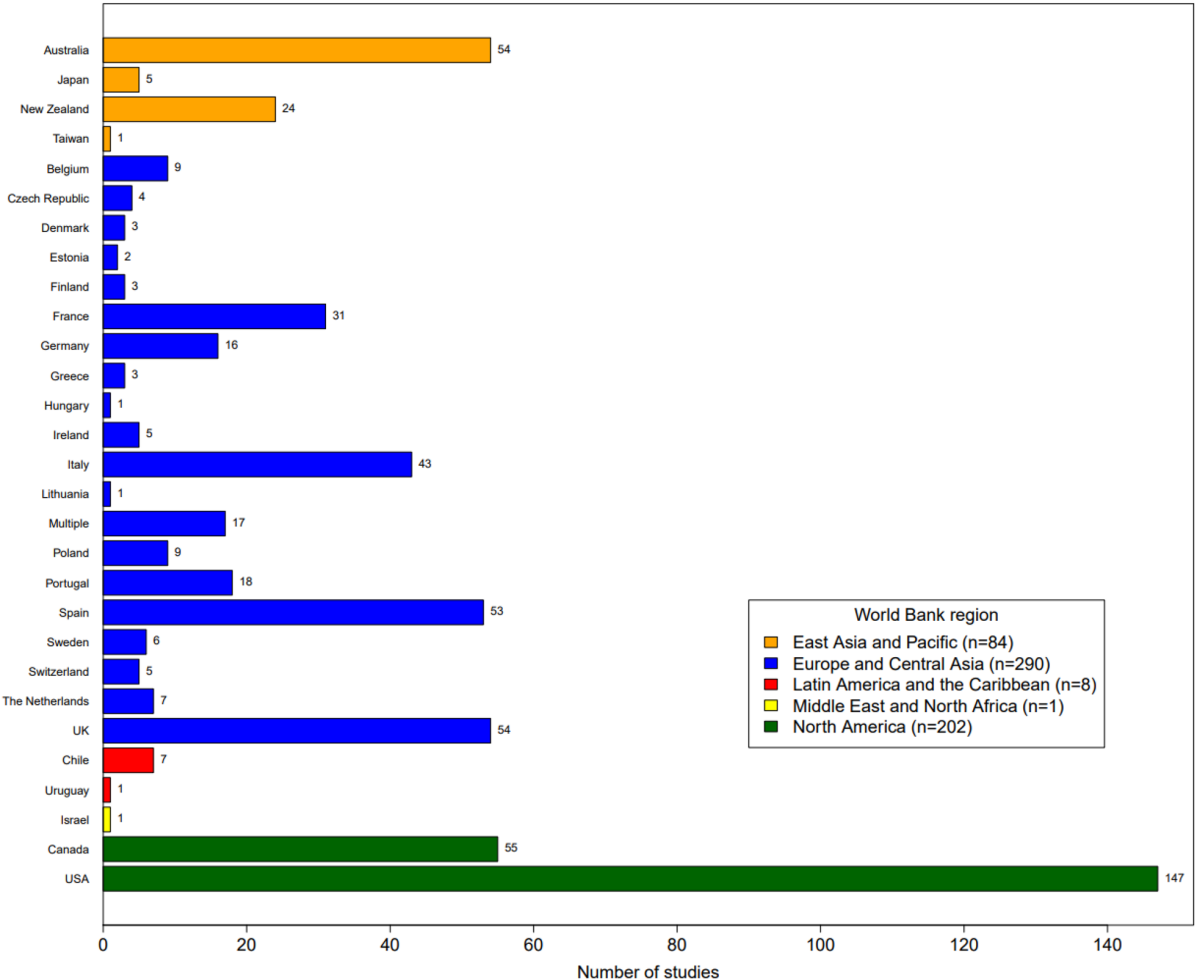
Distribution of primary studies by country/lending group, per the World Bank classification system

This SM includes primary studies from across HICs in different world regions (Figs. 3, 4). Europe (or, Europe and Central Asia, per the World Bank country and lending group region classification) was the most studied region with 290 studies (50%). The second most studied region in this SM was North America with 202 studies (35%), followed by East Asia and Pacific with 84 studies (14%). Eight studies were conducted in Latin America and the Caribbean (1%), and one was conducted in the Middle East (or, Middle East and North Africa, per the World Bank country and lending group region classification) (< 1%).

Within the regions, countries were unevenly represented. By far, the most studied country is the United States (n = 147, 25%). In Europe, the most studied countries were in Spain (n = 63, 11%), the United Kingdom (n = 61, 10%), Italy (n = 49, 8%), France (n = 38, 6%), and Portugal (n = 26, 4%), while many other countries/lending groups had few to no studies. Canada and Australia were also both hubs of agroforestry research, with 55 and 54 studies, respectively (9% each). Of the three major climate zones, the temperate region dominates this study, as most HICs are in the temperate zone (Fig. 3).

### Practices and outcomes evaluated within primary studies

**Fig. 5:**
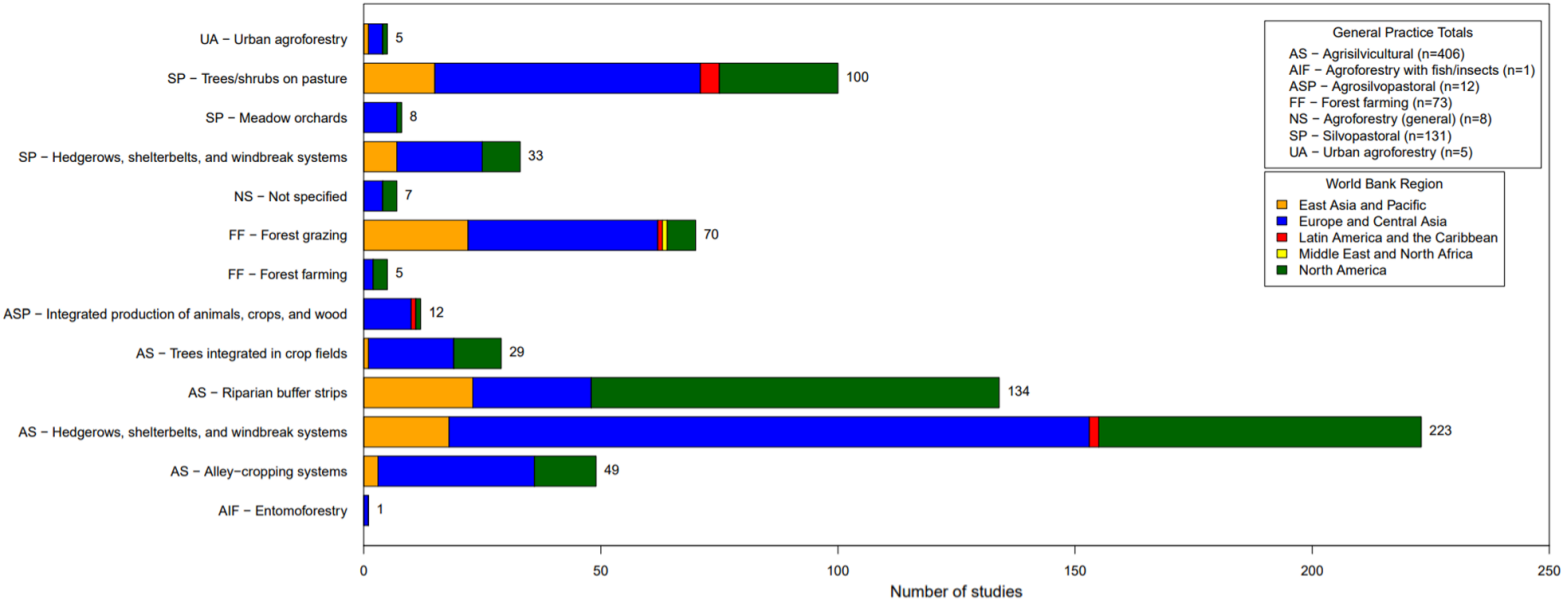
Distribution of primary studies by specific agroforestry practice, by region, and with totals for general practice types

As discussed above, we identified six different general practice types, representing fourteen different specific practices. Figure 5 shows the distribution of studies by agroforestry practice described, noting that some articles looked at more than one type of agroforestry practice. The most common general practice type was agrisilvicultural. Over two-thirds of the studies evaluated agrisilvicultural practices (n = 406, 69%). The second most common practice was silvopastoral agroforestry, with 131 studies (22%). Forest farming was described in 73 studies (12%) and agrosilvopastoral practices were analyzed in 12 studies (2%). Only five studies evaluated urban agroforestry (< 1%) and one study considered agroforestry with fish/insects (< 1%). Eight of the studies (1%) did not specify what they meant by agroforestry, which we marked as agroforestry (general). Note that the total percentage here, and at different points throughout this report, can be more than 100% as a given study could include more than one practice/intervention and outcome. The percentages throughout are based on the percent of studies evaluating the specified subject.

When looking at specific practices (Fig. 5), hedgerows, shelterbelts, and windbreak systems surrounding crop fields or pasture were by far the most studied (n = 256, 44%; n = 223 for crop fields, 38%, n = 33 for pasture, 6%). The next most studied practices were riparian buffers (n = 134, 23%) and trees/shrubs on pasture (n = 100, 17%). Most of the forest farming studies looked at forest grazing (n = 70, 12%), with few considering forest farming (n = 5, < 1%). Alley cropping was studied in 49 studies (8%), and trees integrated in crop fields was studied in 29 studies (5%). Although we found more studies looking at forest farming and at urban agroforestry, few had a relevant comparator, resulting in only five included for each of these practices.

By world region, North America disproportionately studied riparian buffers, and this was the most studied practice within the region (n = 86), followed by hedgerows, windbreaks, and shelterbelt systems (n = 76). East Asia and the Pacific had relatively more studies on forest grazing and silvopasture (n = 45), having the same number of studies on these systems as on agrisilvicultural systems (n-45). Europe dominantly studied hedgerows, windbreaks, and shelterbelt systems (n = 153), followed by trees on pasture (n = 56) and forest grazing (n = 40).

**Fig. 6:**
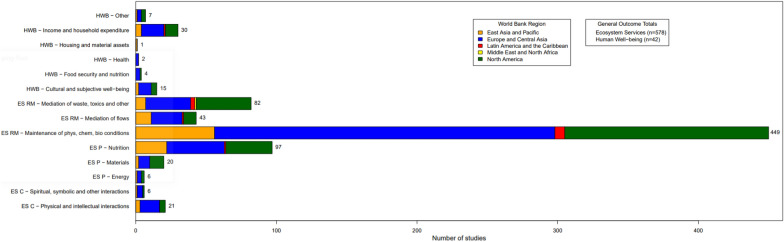
Distribution of primary studies by specific outcome, by region, and with totals for general practice types

Figure 6 shows the distribution of studies by agroforestry outcomes assessed. Ecosystem services is by far the more studied general outcome category (n = 578, 98%) followed by human well-being (n = 42, 5%). The most common specific outcome studied was the regulation and maintenance of physical, chemical, and biological conditions (n = 449, 77%), an ecosystem services outcome. The next most common outcomes were provisioning of nutrition (agricultural production of food) (n = 97, 17%) and regulation and maintenance for mediation of waste, toxics, and other nuisances (n = 82, 14%). The human well-being outcome category was much less studied. The most common outcomes for the human well-being category were income and household expenditure (n = 30, 5%) and cultural and subjective well-being (n = 15, 3%).

By world region, across the board, regulation and maintenance of physical, chemical, and biological conditions was the most studied practice (Europe n = 242, North America n = 145, East Asia and Pacific n = 56, Latin America n = 7). Provision of nutrition was generally the next most studied outcome for all regions (Europe n = 41, North America n = 33, East Asia and Pacific n = 22, Latin America n = 3). North America had slightly more research on regulation of wastes, toxics, and other nuisances (primarily, carbon sequestration and nutrient filtration) (n = 39) and this was the outcome studied in the only included study conducted in the Middle East.

**Fig. 7:**
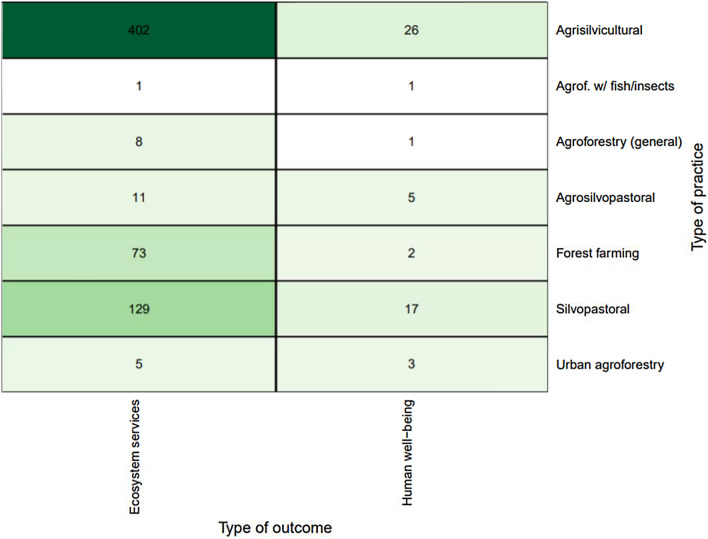
Distribution of primary studies by practices and outcomes (darker green indicates higher concentration of evidence)

Looking at the combination of practices and outcomes, Fig. 7 shows that the majority of practice-outcome linkages focus on agrisilvicultural practices and examine impacts on ecosystem services (n = 402, 69%). The second most common combination of practice and outcome is silvopastoral and ecosystem services (n = 129, 22%). Another concentration of evidence was forest farming and ecosystem services (n = 73, 12%) and agrisilvicultural practices and human well-being (n = 26, 4%). There were relatively few observations focused on human well-being outcomes, but there were 26 studies on human well-being impacts of agrisilvicultural practices (4%) and 17 for silvopastoral practices (3%).

**Fig. 8:**
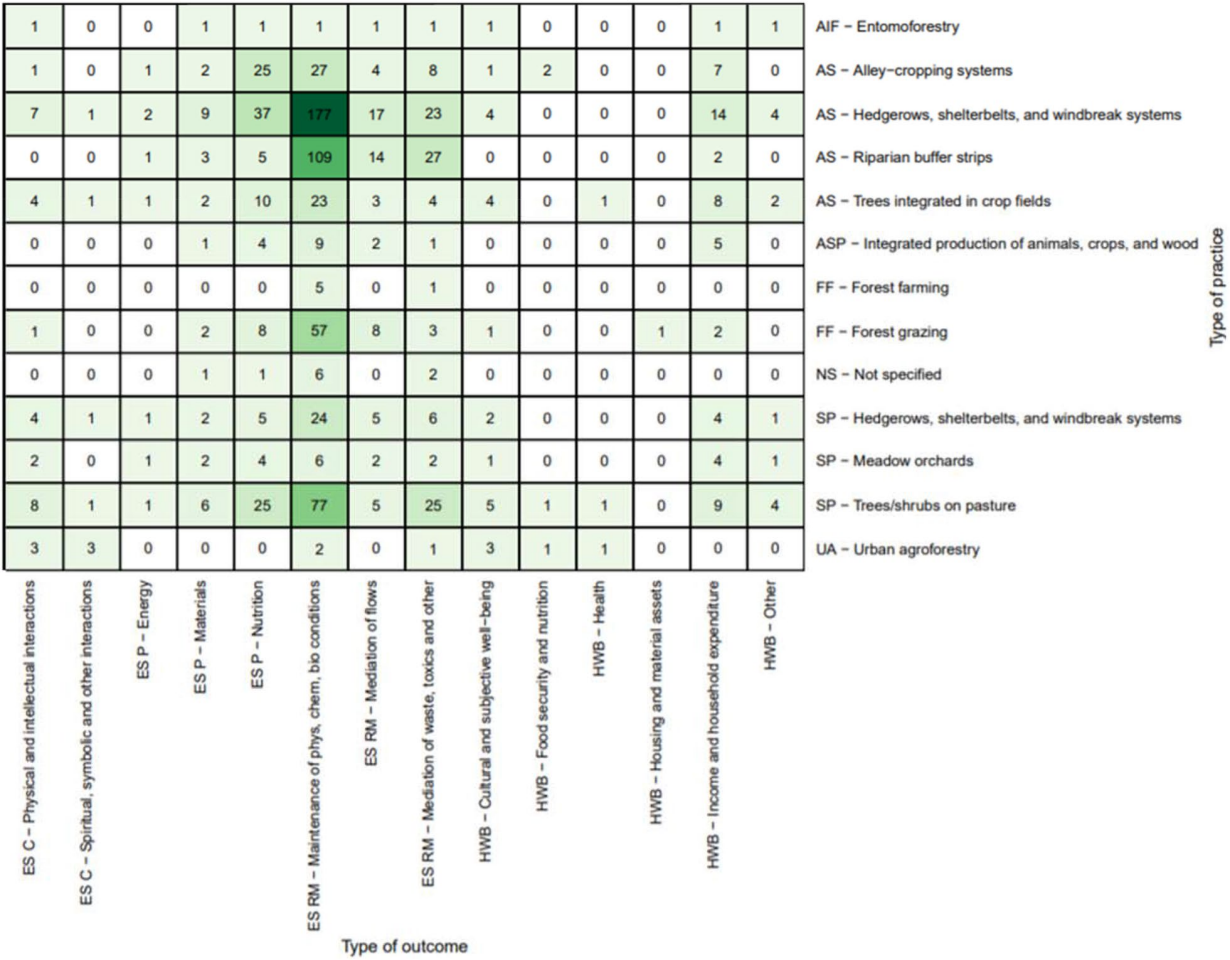
Distribution of primary studies by specific practices and outcomes (darker green indicates higher concentration of evidence)

Figure 8 shows the diversity of more specific linkages between practices and outcomes. The most studied linkage was the effect of hedgerows, shelterbelts, and windbreak systems on the regulation and maintenance of physical, chemical, and biological conditions (n = 201, 34%). These studies are primarily ecosystem services related to biodiversity/habitat provision and soil and water quality. The other most studied linkages were for studies that focused on this same outcome with riparian buffer strips (n = 109, 19%), trees/shrubs on pasture (n = 77, 13%), and forest grazing (n = 57, 10%).

This heat map reveals a concentration of studies on the impacts of linear boundary practices (hedgerows, shelterbelts, windbreaks, riparian buffers) on ecosystem services such as habitat provision for biodiversity and for soil and water conservation. At the same time, it shows some major gaps, with many linkages poorly explored or not examined at all. Although there is some evidence of on-farm impact of agroforestry practice on yields, much of this information comes from field trials conducted at experimental stations, which were not included in this SM. We identified very little evidence on human well-being impacts, such as those relating to health, nutrition, and income. Among ecosystem services outcomes studied, our map reveals a focus on regulating and maintenance rather than provisioning or cultural services.

### Concentrations and gaps in the literature on agroforestry practices and outcomes

We found a lack of studies on several types of practices and outcomes. On the outcomes, there is a lack of studies on the human well-being impacts of agroforestry. While agroforestry is promoted for many social and environmental services, the literature strongly focuses on the environmental outcomes. Regulation and maintenance of physical, chemical, and biological conditions, largely consisting of soil and water quality, carbon storage, and biodiversity outcomes, was by far the most studied outcome for both this SM for HICs and the L&MIC EGM. Health, housing and material assets, and food security and nutrition outcomes for the human well-being category were especially poorly covered. There was more of a focus on cultural and subjective well-being outcomes in HICs. These cultural drivers were found to be a significant motivator for practicing agroforestry in HICs. Compared to the L&MIC EGM, we found more evidence on the cultural and subjective measures of human well-being, such as aesthetic and cultural value, but less evidence on food security and nutrition.

One limitation of the evidence on ecosystem service outcomes is that much of the literature focuses on on-farm and local impacts of agroforestry practice, with few studies considering the effects of agroforestry at the landscape scale (both the impacts of an agroforestry-based landscape and the impacts of agroforestry practices across the landscape). Additionally, although there was considerable literature on the impacts of agroforestry on carbon sequestration, there was a surprising lack of evaluation of agroforestry for climate change adaptation and mitigation. Agroforestry is broadly promoted as a potential pathway to help as a climate change solution through system resiliency and diversification; however, there was little evidence supporting this that met our criteria as an impact study. This lack of research is likely in part due to the current low rates of adoption, which limits researchers’ ability to perform large-scale analyses of systems under extreme climate events, for instance.

Surprisingly, there was limited research on the profitability of agroforestry systems in HICs. This is a key gap in our understanding when we consider the barriers to agroforestry adoption. There is likely a difficulty in capturing the productivity and profitability dimensions of agroforestry systems since these systems take a long time to mature, requiring substantial, long-term data beyond typical funding cycles. The long lifecycle and system complexity represent challenges in collecting reliable long-term data on productivity and profitability. There is also high variability in the design of agroforestry systems and the comparative productivity and profitability outcomes are highly dependent on location and climate variables, such as precipitation and occurrence of extreme weather events. However, researchers have been able to study these dimensions to some extent, though more so for linear boundary plantings, which are often relatively simple, compared to more diverse production systems, such as perennial polyculture plantings.

The practice with the least amount of evidence is that of agroforestry including insects and fish (entomoforestry and aqua-silvo-fishery). We found only two studies that considered this topic that met our inclusion criteria. We expect that this agroforestry practice is not especially prevalent on farmers’ land, which explains why it has not been studied much. Furthermore, several studies on this topic that met most of our inclusion criteria did not include a relevant comparator and were therefore excluded. The second least researched category of practices is agrosilvopastoral, which we expect may be more prevalent in the world and may be more deserving of further investigation. There was also a surprising lack of studies on orchard meadows, given that these types of systems are likely more profitable to farmers and widely practiced, particularly in Europe. Finally, there was little evidence for urban agroforestry. This is in part due to our comparator criteria, as several studies on urban agroforestry systems took inventory of the species and nutrition provided by those systems without providing any relevant comparator for those outcomes.

### Characteristics and trends of the evidence base from primary studies on agroforestry policy interventions

Of the 585 articles concerning primary studies, only 33 included some discussion of policy interventions (see Table 2) promoting the adoption of agroforestry practices (6%), and only one of these studies used a quasi-experimental method to evaluate the impacts of the policy interventions. This study used exposure to a policy intervention, the Great Plains Shelterbelt Project in the USA, as an instrumental variable to evaluate the short- and long-term effect of tree planting programs on agricultural revenue [62] (later published, [63]). We found no policy intervention studies that used a randomized experimental design. Overall, there is an apparent lack of impact evaluations for agroforestry programs and policies in HICs.

These articles were published between 2005 and 2018, with no studies concerning agroforestry interventions published between 2000–2004 or 2019–2020. The years with the most publications concerning agroforestry interventions were 2015 (n = 7, 21%), 2018 (n = 6, 18%), and 2009 (n = 4, 12%). Most of the articles were published as journal articles (n = 29, 96%), three were organization reports (9%), and one was a PhD dissertation. The countries with the most policy intervention studies were Italy (n = 7, 21%), UK (n = 8, 24%), and USA (n = 9, 27%). Sweden had three policy intervention studies (9%), Switzerland had two (6%), and one study was found for each Canada, New Zealand, Poland, and Spain (3%, each).

Incentive provision was by far the most common policy intervention type (n = 26, 79%). The most studied practices for all policy interventions were agrisilvicultural practices (n = 24, 73%), with the most common types of specific practice being hedgerows, shelterbelts, and windbreak systems (n = 19, 58%). The most studied policy intervention-practice linkage was incentive provision with hedgerows, shelterbelts, and windbreak systems (n = 18, 55%). By far, the most studied outcome was ecosystem services (n = 29, 88%). Of these, the most studied specific outcome was regulation and maintenance of physical, chemical, and biological conditions (n = 24, 79%). The most studied policy intervention-outcome linkage was incentive provision with regulation and maintenance of physical, chemical, and biological conditions (n = 22, 67%).

A notable finding of our review is that community-level campaigning/advocacy and market linkage facilitation are not the subjects of any impact evaluations. There is a paucity of such policy intervention impact studies generally, with only incentive provision moderately studied in HICs. We found more studies on farmer capacity development than we included since many of these studies only look at adoption of agroforestry, without looking at the subsequent social-ecological outcomes.

### Concentrations and gaps in the literature on agroforestry policy interventions

We identified several gaps in the existing agroforestry policy intervention literature. This study revealed that rigorous evidence on the effects of agroforestry policy interventions remains extremely limited. The included policy intervention studies primarily consisted of evaluations of incentive provision type interventions, including payments for implementing conservation practices. We found little evidence on the impacts of other types of policy interventions. This finding is especially surprising for farmer capacity development interventions since there are many programs in HICs designed to provide technical support and training to farmers on agroforestry, which represents substantial investments by governments and agencies. The focus of the limited research on farmer capacity development for agroforestry practices in HICs tended to only consider adoption as an outcome, which was not included in this map. This SM suggests that there is a crucial need to improve the evidence base on this topic, particularly with respect to specific policy interventions.

In terms of policy intervention outcomes, regulation and maintenance ecosystem services were generally well-studied, with significantly less work studying the impacts of agroforestry policy interventions on human well-being outcomes. This is strikingly different from the findings from the L&MIC EGM [16] and systematic review of L&MIC agroforestry policy interventions [24], which found relatively little work evaluating the impacts of agroforestry policy interventions on environmental impacts, with a focus on agricultural productivity and human well-being instead. There was also more literature on the impacts of agroforestry policy interventions in L&MICs, with 40 studies evaluating agroforestry policy interventions between 2000 and 2017, eleven of which were impact evaluations using quasi-experimental methods [24]. This highlights a major difference in the types of objectives for agroforestry between HIC and L&MIC income groups. L&MICs are often the target for international aid to promote the UN Sustainable Development Goals, with a focus on human livelihood and food security [2, 10]. On the other hand, the main objective of agroforestry in HICs has thus far tended to be the integration of conservation practices into agricultural lands.

There are several likely reasons for these gaps in research on agroforestry policy interventions. First, agroforestry sits at the intersection of agriculture and forestry, and balances between production and conservation services, and this has often meant that the research communities of each field neglect agroforestry. This grey area where agroforestry falls also means that agroforestry is often overlooked by policies, as currently most countries have separate agricultural policies and forestry policies without national agroforestry policies. Second, there is often a significant lag between the adoption of agroforestry practices or systems, and measurable outcomes. Therefore, a complete evaluation requires a long-term commitment that increases the cost and time requirements of such studies.

### Characteristics and trends of the evidence base from systematically conducted reviews

We identified 41 systematically conducted reviews (systematic maps, systematic reviews, meta-analyses that used systematic searches, and ongoing systematic map/review protocols) that fit the inclusion criteria. Only three of the identified reviews, [64–66], included evidence relating to agroforestry policy interventions. Additional file 5 provides detailed information on each of the 41 systematically conducted reviews (SCRs) included. Many of the included reviews discussed multiple practices and outcomes. The SCR articles were published between 2006 and 2020, with none published between 2009 and 2011 and only seven (17%) published between 2006 and 2014. SCRs for agroforestry may therefore be a more recent trend. Seven articles were published in 2020 by the time of our search (17%), four in 2019 (10%), ten in 2018 (24%), four in 2017 (10%), three in 2016 (7%), and six in 2015 (15%). Of the 41 articles, 18 were classified as meta-analyses using a systematic literature search (but not following other systematic review guidelines) (44%), 13 were systematic reviews (which often presented meta-analysis results) (32%), seven were systematic maps (17%), and three were in-progress systematic map/review protocols (7%). Ten of the included systematic studies used a protocol published prior to conducting their review (24%). There was a notable concentration of work focused on European countries, with several systematic studies published under the AGFORWARD project in the EU. Almost half of the reviews (n = 19, 46%) were global in scope, covering temperate, subtropical, and tropical climates. All the included studies used a systematic search strategy, but we also identified over 200 additional review-type articles that did not use systematic search strategies and therefore were not included in the SM.

**Fig. 9:**
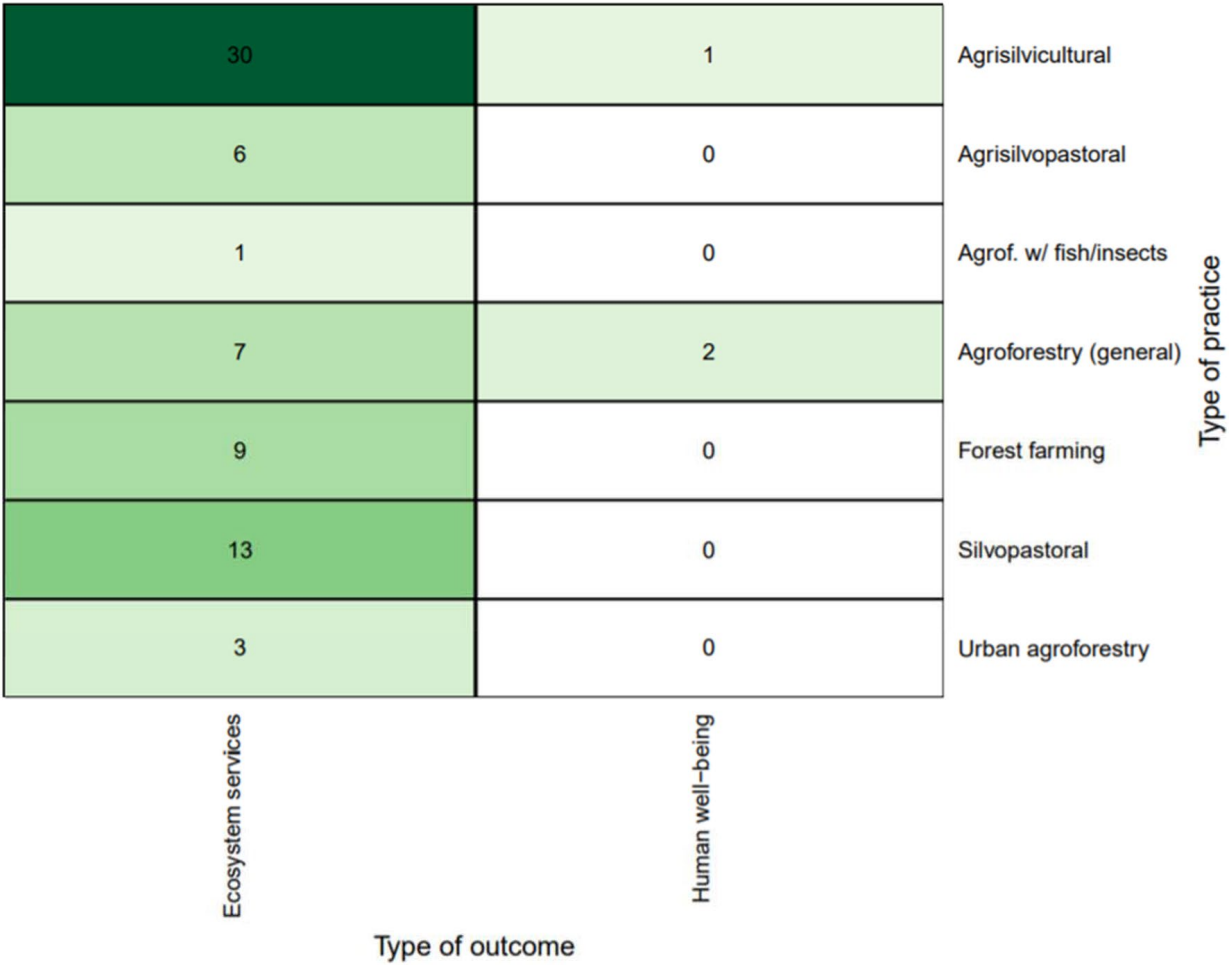
Distribution of systematic reviews by practices and outcomes (darker green indicates higher concentration of evidence)

Figure 9 summarizes all data found by number of linkages between each general practice category and outcome type considered by the reviews. If one review stated a practice with two different outcome types or a single outcome with multiple practices it would be counted multiple times in Fig. 9. Like the findings for the empirical evidence, agrisilvicultural practices were the most common agroforestry practice studied (n = 30, 73%) followed by silvopastoral (n = 13, 32%). Nine studies looked at forest farming practices (22%), six at agrosilvopastoral practices (15%), one study considered agroforestry with insects/fish (2%), and three considered urban agroforestry (7%). Nine other studies did not specify any specific agroforestry practice (22%). For the types of outcomes evaluated by the SCRs, ecosystem services were the most common outcome studied (n = 39, 95%). Only three studies considered human well-being as an outcome in their review (7%).

**Fig. 10:**
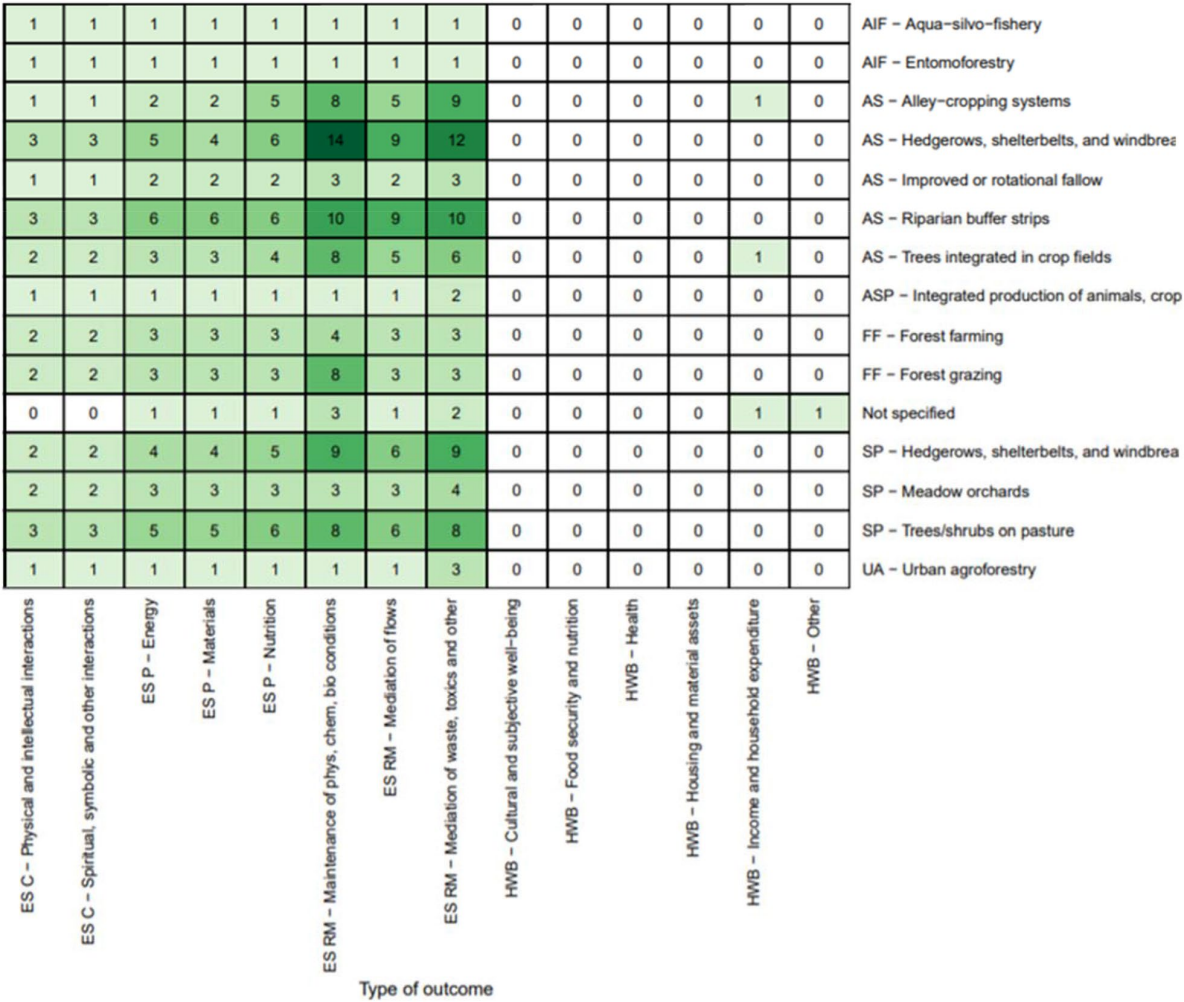
Distribution of systematic reviews by specific practices and outcomes (darker green indicates higher concentration of evidence)

Figure 10 details the specific breakdown of the practices and outcomes. Regulation and maintenance of physical, chemical, and biological conditions was the most common outcome type (n = 29, 71%). Twenty studies considered regulation and maintenance to mediate wastes, toxics, and other nuisances (49%) and 15 to mediate flows (37%). Thirteen (32%), eight (20%), and eight (20%) studies considered the ecosystem services of provisioning of nutrition, energy, and materials, respectively. Four reviews considered the multiple dimensions of cultural ecosystem services (10%). For the three reviews that considered human well-being, two (5%) evaluated income and household expenditure and one (2%) considered other dimensions of human well-being.

Among the specific practices from Fig. 10, hedgerows, shelterbelts, and windbreak systems on arable fields was the most studied (n = 20, 49%), followed by riparian buffer strips (n = 15, 37%), hedgerows, shelterbelts, and windbreak systems surrounding pasture (n = 15, 37%), and alley-cropping systems (n = 14, 34%). There were 13 studies that considered trees/shrubs on pasture (32%) and 11 that looked at trees integrated in crop fields (27%). Nine studies looked at forest grazing (22%), and five considered forest cropping (12%). Five considered improved or rotational fallow (12%), five on meadow orchards (12%), three urban agroforestry (7%), two for integrated production of animals, crops, and wood (5%), one for aqua-silvo-fishery (2%), one for entomoforestry (2%), and six not specified (15%).

### Concentrations, gaps, and opportunities in the literature synthesizing agroforestry research

We identified many systematically conducted review studies that can inform policy immediately. We also identified concentrations of evidence for further systematic review of available evidence. This SM offers a tool to identify literature to systematically review the literature globally on many of these topics. We found 41 completed systematic reviews, systematic maps, and meta-analyses, but these do not cover all the concentrations of evidence or cover all the global regions. Generally, the concentration of existing evidence syntheses largely reflects the concentrations in primary evidence; however, as noted previously, these gaps in economic and social outcomes may be key to understanding the low rates of agroforestry adoption in HICs.

There are important research and synthesis gaps on the impacts of agroforestry on social outcomes, particularly economic and human well-being outcomes. For areas with minimal literature available per our inclusion criteria (e.g., health, food security and nutrition, and housing and material assets), evidence synthesis would not yet be feasible. On the other hand, outcomes such as economic well-being (income and expenditure outcomes) are more well-studied and offer a relevant area for additional evidence synthesis. Recent work is offering some synthesis of economic outcomes of agroforestry, e.g., the work by Jordan et al. [31], which included enterprise economics in their systematic map on silvopastoral systems. The impact of agroforestry practices on human well-being is clearly an important factor in people’s willingness to pay for and willingness to adopt agroforestry. However, much of the existing evidence is based on observational and survey/perception studies. Additionally, evidence synthesis is needed on the impacts to agricultural productivity, as well as the impacts on agroforestry on income and household expenditure. These syntheses may need to derive data from field trials not included in this SM as well as the on-farm studies included in this SM, as much of the literature on yield and economics comes from field trials and there is as yet limited evidence from on-farm studies on these outcomes.

We found that there was a clear concentration of evidence on linear boundary plantings (hedgerows, shelterbelts, windbreaks, and riparian buffers), including several systematic meta-analyses, e.g., [64, 67–71]. Linear boundary plantings are typically along the edge of fields and do not interfere with conventional agricultural practices. There is substantial evidence on several outcomes for these linear boundary plantings, especially for biodiversity and habitat provision, runoff and erosion, soil and water quality, and carbon sequestration as well as on crop yields as a function of distance from the boundary plantings. These areas represent possibilities for further evidence synthesis and review to better understand the impacts of linear boundary plantings, as also highlighted in [39] and [72]. These practices may be highly suitable to incorporate into HIC agriculture for environmental benefits and at a lower cost than other agroforestry practices.

There was also a concentration of evidence for trees with livestock—both silvopastoral practices and forest grazing practices. One area for potential systematic review is on the relative advantages and disadvantages between different practices integrating trees and livestock, i.e., planting trees into pasture versus thinning trees from forests for grazing. There is substantial evidence for both types of practices, and further evidence synthesis of this literature could contribute towards understanding the differences in productivity and ecosystem services based on land use history. Some work has already been done on systematically reviewing the literature on forest grazing, as in [73, 74], and [75]. A better understanding of the differences between silvopasture and forest grazing could help extension agents make recommendations to farmers.

While we did not extract information on scale for our SM, the impacts of implementing agroforestry practices at different scales (e.g., agroforestry practices implemented at the plot, farm, or landscape scale) would be a useful area for future evidence synthesis. Similarly, evidence synthesis considering the scale of the impact of agroforestry practices, i.e., how agroforestry effects ecosystem services and human well-being outcomes on the farm and across the landscape, would be another key area for future synthesis. However, there is currently limited evidence on the effects of agroforestry at the landscape scale, which would limit syntheses considering scale.

There is also room to synthesize evidence from more world regions. The bulk of evidence synthesis efforts have focused on Europe (notably, [3] and [38]) or globally. This suggests a potential for more region-specific evidence synthesis, such as across North America or the Pacific (e.g., Australia and New Zealand). For instance, there is a prevalence of studies on the ecosystem service impacts of agroforestry in the USA, but little attention has been given towards synthesizing this body of literature, in contrast to the literature from the EU that has been subject to much more evidence synthesis.

### Limitations of the map

A limitation of this map is that it only included English language articles. There may be a large amount of agroforestry research that is missed by the larger body of agroforestry literature as it is published in languages other than English. For example, there may be several relevant articles published in French, Portuguese, Spanish, or Japanese that we did not include due to the language restriction. There has been significant investment in studying dehesa and montado systems and associated policies in Spain and Portugal, but likely not all the research was published in English. There has also been strong interest by the French government in agroforestry, with many field trials through the Institut national de la recherche agronomique (INRA), such as the Restinclières Agroforestry Platform, as well as agri-environmental schemes in France intended to support agroforestry, and the impacts of this work may in part have been published in French rather than English.

In this study, we found that many seemingly relevant articles were not included based on our criteria. Many studies, particularly those on agroforestry policy interventions, tended to stop their analysis at adoption of agroforestry without continuing to assess the subsequent social-ecological impacts. Many studies also lacked a non-agroforestry control to compare the relative impact of different land uses, which is vital for establishing convincing arguments for the impacts of agroforestry. We did not include simulation-based studies with predictive models since those do not evaluate the realized real-world impacts, even though these types of studies can make important contributions in this field. Finally, the SM did not include agroforestry field trials conducted at experimental stations since they did not show the direct outcomes realized by farmers, which was our population of interest. The list of potential research station field trial studies is available in Additional file 6.

Additionally, we recognize the connection between ecosystem services and human well-being. The relationship between ecosystem services and human well-being outcomes is complex, with distinct differences and areas of overlap [76, 77] While ecosystem services and human well-being outcomes are inherently linked, it is possible to examine them as separate outcomes, as we have done here and as others have done in many other studies. Separating these outcome categories is done to improve clarity of presentation, capture the breadth of social and ecological research on agroforestry, and target a broader audience, including those more or less interested in ecosystem services versus human well-being outcomes. Although the ecosystem services framework and human well-being framework are largely separate, we acknowledge some overlap in cultural ecosystem services and human well-being outcomes. When a study fell into both outcome categories, the study was classified in both categories.

Furthermore, we acknowledge that these frameworks are continually evolving. We developed our definitions and scope of this SM in 2017–2018, when the protocol was published and searches were conducted, so more recent developments in these fields are not fully captured in this SM. For example, we did not incorporate the term “nature’s contribution to people” into our SM. However, the use of the term “ecosystem services” largely, if not fully, covers “nature’s contribution to people,” and we do not believe this choice substantially affects our SM results.

The study of system scale, specifically, is beyond the scope of this SM. This is an interesting area for future study and would be interesting to examine more closely in a systematic review. We included studies that considered any system scale (plot, farm, landscape, etc.) that implemented agroforestry practices, i.e., we did not seek to limit our map by scale. We took an approach of inclusivity for this SM, where we chose to include studies with debatable relevance (e.g., studies with questionable study quality), as the scope of the SM is intentionally broad so as to present as comprehensive a picture as possible of available evidence on this topic. We strictly followed our PICO guidelines for all studies, regardless of scale of analysis. We did not attempt to infer results beyond what was presented in the study.

## Conclusions and implications

A central finding of this review is that, while there are hundreds of observational studies on agroforestry practices, the evidence base on the impacts of agroforestry policy interventions remains very thin. The results of this SM highlight those areas where greater concentrations of evidence exist while also revealing a number of important gaps in evidence.

We note that there are two main reasons practice-outcome linkages may have little or no evidence: (1) the linkage is of research and policy interest but has not been well studied, or (2) the linkage is not of significant research and policy interest, including cases where the practice or policy intervention does not link logically with a given outcome, and therefore has not been investigated. Below we draw out implications of these overall findings for research, policy, and practice. In examining both practices and policy interventions, this report provides a comprehensive portrait of the current evidence on the impacts of agroforestry.

### Implications for policy/management

As a result of this work, we have identified a few key implications for policy/management. From a donor perspective, this SM highlights major areas where there is a need to support more primary research, particularly on specific kinds of agroforestry policy interventions, as well as where evidence synthesis might be conducted. Relatedly, there is a major opportunity for donors, governments, and other partners to work together to support and implement experimental and quasi-experimental studies of different agroforestry policy interventions to enhance our understanding of what works and what does not seem to work in this area. Without more reliable evidence on policy intervention pathways and impacts, agroforestry risks further marginalization, thereby undermining progress on broader development and sustainability goals.

### Implications for research

The most notable gaps relating to studies of agroforestry policy intervention impacts are the low number of studies overall and, specifically, those using experimental or quasi-experimental impact evaluation methods. Agroforestry is increasingly promoted and supported by agencies worldwide, yet there exists little evidence of the impacts of this support. Only one of the 33 policy intervention studies in this SM was an impact evaluation that used quasi-experimental designs, and no policy intervention study used a randomized control trial (RCT) approach. Such impact assessment is clearly possible, however: this SM includes 76 on-farm experimental studies and 30 before-after studies of agroforestry practices. Further studies on the impacts of agroforestry practices are still needed, but there is a particularly strong need for carefully designed impact evaluations using experimental or quasi-experimental designs. Such studies can help identify what types of policies and programs supporting the adoption and implementation of agroforestry practices work, where, why, and how.

The complexity that comes with the integration of agricultural, forestry, and pastoral practices, as is the case in agroforestry, poses significant challenges to evaluating the effectiveness of specific agroforestry policy interventions. However, given the potential for agroforestry to contribute to multiple environmental and rural development goals simultaneously, there is an urgent need for such impact evaluations. And existing evaluations (e.g., [24, 63]) show that such evaluation is possible to expand and improve the current evidence base.

While the literature has focused on regulation and maintenance type ecosystem services as an outcome, the under-studied social dimensions are likely critical in explaining why agroforestry is not more widely adopted. Understanding social and economic outcomes may shed light on the reasons for the currently low rates of agroforestry adoption in HICs and help understand what types of programs and policies are necessary to advance the implementation of agroforestry practices. There is considerable literature on the motivations and barriers for farmers in HICs adopting agroforestry [78–82]. Many studies in HICs have found that there is a lack of knowledge regarding the different agroforestry practices and a lack of technical knowledge on how to effectively implement such practices. There are also financial barriers with the long lifecycle of trees on farms and attendant risks to long-term success of the system for the farmer. Although the environmental benefits of some agroforestry practices are generally well understood by farmers, these barriers prevent them from choosing to implement agroforestry practices on their farmland. To address the disconnect, knowledge of the on-farm social and economic impacts of agroforestry is vital. It is necessary to consider and address the costs and barriers to agroforestry adoption when designing programs and policies [80, 83, 84].

Geographically, there were clear concentrations of evidence in the USA, Spain, the UK, Italy, France, Canada, and Australia for studies on agroforestry practices. Together, these seven countries were the focus for 80% of the studies on agroforestry practices in HICs, with the USA alone accounting for 25% of the included studies. These results are partially due to the size of the United States, with studies spread out across many states, and because we did not include field trials conducted at experimental stations. Regionally, Europe has the largest number of studies; however, 15 of the 37 HICs in Europe had no studies specifically conducted within them (though it is likely that many of the studies conducted in neighboring countries could be relevant to those countries). We also found that in total 48 of the 79 HICs had no studies conducted within their borders. This represents a potential gap in the literature and a potential limitation of this study, which only searched English language sources. Studying agroforestry practices in underrepresented countries offers an opportunity in agroforestry research.

One area within the USA that drew our attention is the USDA Sustainable Agriculture Research and Education (SARE) grant reports. We included nine completed reports along with six in-progress studies from SARE. Many of the SARE-funded studies were conducted on-farm with the study design and implementation run by the farm manager; other on-farm SARE studies had extension agents or advisors from academia assisting at different points of the study. There were also SARE-funded studies that are primarily conducted by professors/researchers but carried out on private farms. Overall, there is clearly an effort from SARE to engage farmers and other agricultural professionals with agroforestry information and practices. There are also many outreach interventions throughout the SARE grant list. However, the reporting standards for SARE grants are limited, meaning that many relevant studies were not included in this SM, even though there was an abundance of on-farm agroforestry research funded through this program. It appears that SARE grants are increasing farmer capacity and knowledge, but these grants are not necessarily studying the effectiveness of agroforestry practices. The SARE goal is to increase agroforestry practice and education, rather than evaluate what methods are best at disseminating information to promote agroforestry adoption. Future research on the impacts of SARE projects could help inform agroforestry policy interventions.

Finally, we found a lack of equity focus in the literature, both for policy interventions and for practices, with no studies disaggregating the impacts of agroforestry policy interventions by gender, socio-economic level, race/ethnicity, or literacy/educational level. Only four studies that focused on agroforestry practices disaggregated results by at one or more equity measures. Given the predominant focus on ecosystem service outcomes, which emphasize biophysical impacts rather than social ones, the limited evidence on equity was expected. Nevertheless, there remains a critical need for more impact evaluation studies generally and specifically for those that attend to issues of equity.

## Supplementary Information

**Additional file 1**. List of High-Income Countries and Lending Groups (according to the 2018 World Bank classification [15]).

**Additional file 2**. ROSES for Systematic Map Reports. Version 1.0.

**Additional file 3**. This file contains the exact search strings used, dates of searches, and number of articles returned for each bibliographic database searched.

**Additional file 4**. This file contains the list of websites, the URLs, the number of articles identified, and notes on the search and screening process from relevant organizations that were searched for the grey literature search.

**Additional file 5**. This file contains the data extraction spreadsheet with information on each of the included studies for the high-income country systematic map of the impacts of agroforestry.

**Additional file 6**. This additional file contains the list of excluded studies from the full text screening.

## Data Availability

All data generated or analysed during this study are included in this published article and its Additional files.
